# Family support group in psychosocial rehabilitation

**DOI:** 10.4103/0019-5545.55941

**Published:** 2005

**Authors:** L. Ponnuchamy, Baijumon K. Mathew, Sheeba Mathew, G.S. Udayakumar, S. Kalyanasundaram, Dharitri Ramprasad

**Affiliations:** * Lecturer, The Richmond Fellowship Post Graduate College for Psychosocial Rehabilitation, Bangalore; ** Lecturer, The Richmond Fellowship Post Graduate College for Psychosocial Rehabilitation, Bangalore; *** Lecturer, The Richmond Fellowship Post Graduate College for Psychosocial Rehabilitation, Bangalore; **** Assistant Professor, Department of Psychiatric Social Work, National Institute of Mental Health and Neuro Sciences, Bangalore; ***** Professor, The Richmond Fellowship Post Graduate College for Psychosocial Rehabilitation, Bangalore; ****** Associate Professor, The Richmond Fellowship Post Graduate College for Psychosocial Rehabilitation, Bangalore

**Keywords:** Family support group meetings, psychosocial rehabilitation

## Abstract

**Background:**

Support groups for families of persons with mental illness are emerging as significant components in psychosocial rehabilitation programmes.

**Aim:**

To ascertain the expectations of family members who attend family support group meetings and to find out the efficacy of such programmes.

**Methods:**

The data were collected from support group members using a semi-structured interview schedule. The study sample (*n*=20) was drawn from family members who attended the support group meetings regularly for a minimum period of 6 months. Data analysis was done using percentile.

**Results:**

Analysis of the data revealed that members attending the support group meetings expected to get more information about the illness, develop skills to cope with problems at home and learn skills to deal with the ill person. An important finding of the study was that the members developed a ‘feeling of togetherness’ as a result of being a member of a group with common aims.

**Conclusion:**

Participation in a support group meeting positively affects key variables in the participant's adaptation to mental illness in a relative.

## INTRODUCTION

The role that families play in the support and care of a relative with a mental illness has gained increasing attention over the past 30 years. There has been a rapid propagation of support, self-help, or mutual support groups for family members of persons with severe psychiatric disabilities. In 1979, representatives of 100 family support groups came together to form the National Alliance for the Mentally Ill (NAMI) in the US.^1^ In a 1980 presidential address to the American Psychological Association, Leona Tyler stated that by the year 2000 the standard vehicle for dealing with mental health and health-related psychosocial issues would be self-help support groups.^1^

Family self-help groups are defined as ‘voluntary small group structures for mutual aid in the accomplishment of a specific purpose’. They are usually formed by peers who come together for mutual assistance in satisfying a common need, overcoming a common handicap or life-disrupting problem, and bringing about a desired social and/or personal change.^2^ There are self-help groups for nearly every disease category listed by the World Health Organization, as well as groups concerned with a wide variety of psychosocial problems. There are groups addressing particular psychiatric disorders, such as the Depressives Anonymous, Manic–Depressives Anonymous, Neurotics Anonymous and Schizophrenics Anonymous. There are groups for parents who abuse their children, isolated older people, the handicapped and patients discharged from psychiatric institutions.^3^,^4^

Despite the variation among groups, the sharing of experience among people with a history of a similar problem is the fundamental concept that distinguishes this helping approach from other helping exchanges.^5^ Many assumptions about family support groups have been promoted through the growing literature, as well as through less formal exchanges at conferences and meetings.

In India, the family is the primary caregiver for individuals who have physical or psychiatric illness. The family members of people affected by a mental illness face various problems—financial, emotional, interpersonal relationships, social relationships—affecting the caregiver's day-to-day activities, health and occupation. The families need information, support, knowledge and specific suggestions for coping with mentally ill relatives.^6^ With the increase in de-institutionalization, self-help groups are bridging the gap between hospitalization and community living. Over the past two decades, self-help groups have become an important way of helping people cope with various problems due to mental illness.

There are self-help groups for families of persons with mental illness in India. Most of the self-help groups later developed into associations or agencies to improve the quality of life of caregivers (as most parents are aged) and for advocacy. Hence, the majority of these groups started including significant relatives and others as members, such as the Alliance for the Mentally ill, Chennai, Tamil Nadu; Association for the Mentally Disabled (AMEND), Bangalore, Karnataka; Marghadharshi, Bangalore, Karnataka; Marghadeepthi, Guwahati, Assam; Schizophrenia Awareness Association, Pune, Maharashtra; and Subitcham, Madurai, Tamil Nadu.

The present study was done to find out the expectations of members of a family support group and assess the benefits of family support group meetings.

## METHODS

The study was conducted at Chetana, a day-care centre run by the Richmond Fellowship Society (India), Bangalore. Admission to Chetana is open to any person 18–45 years of age with a diagnosis of schizophrenia or any other major psychiatric disorder or mild mental retardation. The facilities available include vocational training units of computer, typing, printing, plastic moulding, and tailoring and embroidery. In addition to vocational training, the centre has therapeutic programmes such as structured daily activities and afternoon group activities, namely, community meeting, group therapy, recreational activities such as going for movies, picnics, group games and horticulture activities. Regular individual and family therapy sessions, and family support group meetings are also held at the centre.

The present article is a part of the ongoing programme. Hence, some of the tools were not used. As these programmes are service-oriented and ongoing, we are of the opinion that the use of scales is important. The sample consisted of 20 members who had attended the support group meetings regularly for a minimum period of 6 months. A semi-structured interview schedule was used to collect information from the group members to assess the usefulness of such meetings. It consisted of two domains, i.e. expectations and benefits received from the family support meetings along with sociodemographic details (developed by the authors). Each domain includes 4–5 items in the schedule (Box 1).

**Box 1:** Items of the two domains of the interview schedule

**Expectation domain**
Information about the illness Dealing with difficult situations More contact with other families Need for an expert's help
**Benefit domain**
Feeling of togetherness Skills in dealing with the patient at home Emotional support Understanding the problem Reduction of fear and anxiety

The family support group meeting was conducted by the psychiatric social worker regularly every month at the centre. The members of the family support group attended the meeting without fail. The duration of meeting was 60–75 minutes. The format of each meeting included introducing new members to the group, review of the previous month's problems and issues of the psychiatric rehabilitation centre, allowing members to express day-to-day problems faced by the families at home and in society. Family support group members were allowed to discuss in the language known to everyone. Professional interventions such as facilitating ventilation, active listening, providing support, reassurance, psychoeducation, realistic goal-setting, formulating future plans and advocacy were used as and when needed.

## RESULTS

### Sociodemographic profile of the group members

Table 1 gives the sociodemographic profile of the group members. Most of the group members belonged to the age group of 61–70 years (45%). As far as education was concerned, 45% of the group members were undergraduates. Regarding the occupation of the members, most had retired from the services (35%). The majority of the members who attended the group meetings were fathers (55%), 30% were spouses/mothers, 10% were sisters and 5% were brothers. The majority of members (60%) had attended the meetings for more than 6 months (7–9 group meetings) and 40% for a minimum period of 6 months.

**Table 1:** Sociodemographic profile of the group members (*n*=20)

Variable studied	*n* (%)
Age (in years)	
30–40	3 (15)
41–50	3 (15)
51–60	4 (20)
61–70	9 (45)
Above 70	1 (5)
Education	
Up to X (matriculation)	6 (30)
X–XII	1 (5)
Diploma	1 (5)
Undergraduate	9 (45)
Postgraduate	3 (15)
Occupation	
Housewives	4 (20)
Own business	3 (15)
Working in the government sector	2 (10)
Working in the private sector	2 (10)
Professionals	2 (10)
Retired from service	7 (35)
Significant family members	
Father	11 (55)
Spouse/mother	6 (30)
Sister/brother	3 (15)

### Clinical profile of the patients

Table 2 lists the diagnosis of patients and duration of illness. The majority of patients had schizophrenia (80%), the rest of them had mild mental retardation (10%) and affective disorders (10%). Forty per cent had been ill for more than 16 years.

**Table 2:** Diagnosis and duration of illness (*n*=20)

	*n* (%)
Diagnosis	
Schizophrenia	16 (80)
Affective disorders	2 (10)
Mild mental retardation	2 (10)
Duration of illness (in years)	
<5	3 (15)
6–10	7 (35)
11–15	2 (10)
≥16	8 (40)

### Expectations of the group members

The group members had various expectations from the family support group meetings. These are listed in Table 3.

**Table 3:** Expectations of group members (*n*=20)

Expectation	*n* (%)
Information about mental illness	8 (40)
More contact with other families	4 (20)
Dealing with difficult situation	4 (20)
Expert's help	4 (20)

### Benefits of family support group meetings

The benefits of family support group meetings were analysed. The results are presented in Table 4.

**Table 4:** Benefits of a family support group (*n*=20)

Benefit	*n* (%)
Feeling of togetherness	5 (25)
Skills in dealing with the patient at home	4 (20)
Emotional support	3 (15)
Improvement in relationship with the patient	3 (15)
Understanding the problem	3 (15)
Reduction in fear and anxiety	2 (10)

## DISCUSSION

The findings of the study suggested that family members of mentally ill persons with certain demographic characteristics are more likely to join support groups. For example, family members with a higher level of education are likely to join support groups. This finding is consistent with those reported by other authors.^7^–^9^ Although none of these studies found differences between groups in terms of relationship with the disabled relative, our results suggest that more parents join support groups than siblings, spouses or adult children. Our study found that people whose relatives are diagnosed with schizophrenia are more likely to volunteer for research studies than those whose ill relatives are diagnosed with other mental illnesses. This factor seems very interesting and needs further scientific exploration. This consistent pattern indicates a need to find more effective ways of engaging family members dealing with mental illnesses.

Out results support the idea that participation in a support group meeting positively affects certain key variables in the participant's adaptation to mental illness in a relative. In this analysis, members reported more extensive adaptive coping skills and more emotional support and improvement in the relationship. As in the studies by Chamberlin *et al.*^9^ and Carpinello *et al.,*^10^ members reported improvement in the quality of their social network and understanding of the problem.

The results of the present study revealed that the highest expectation of the family members was information about the illness; this is consistent with the findings reported by Elangovan^6^ and Winefield *et al.*^11^ Furthur studies on support groups for families of persons with mental illness could examine the effects on variables that do not seem to have been impacted, such as improvement in the relationship with the patient and reduction in fear and anxiety. Further research could also attempt to explain the mechanism through which support groups have a positive impact on the coping of family members. Perhaps this subsequently improves the ill relative's functioning level and reduces the subjective burden on caregivers. There is a need to conduct long-term studies to understand the process of the group meeting, the dynamics of the interaction, and the utility of such groups. To study the changes that occur in a family we need to conduct experimental studies with a control group.
